# Varenicline and Risk of Self-Harm: A Nested Case-Control Study

**DOI:** 10.1371/journal.pone.0163681

**Published:** 2016-09-23

**Authors:** Mina Tadrous, Diana Martins, Zhan Yao, Muhammad M. Mamdani, David N. Juurlink, Tara Gomes, Tony Antoniou

**Affiliations:** 1 Li Ka Shing Knowledge Institute, St. Michael’s Hospital, Toronto, Canada; 2 Institute for Clinical Evaluative Sciences, Toronto, Canada; 3 Sunnybrook Research Institute, Toronto, Canada; 4 Department of Family and Community Medicine, St. Michael’s Hospital, Toronto, Canada; Legacy, Schroeder Institute for Tobacco Research and Policy Studies, UNITED STATES

## Abstract

**Background:**

Smoking remains a serious public health concern. Pharmacotherapy for smoking cessation, including bupropion and varenicline, are proven means to increase quit rates. Post-marketing reports describing suicidal behaviours have raised concerns about the safety of varenicline. However, whether varenicline imparts a higher risk of suicide relative to bupropion remains uncertain.

**Methods:**

A population-based nested case-control study in Ontario, Canada, from April 1, 2011 to March 31, 2015 was conducted. Subjects were residents of Ontario aged 18 years and older with publicly funded drug coverage receiving either bupropion or varenicline for smoking cessation. We defined cases were those with a hospitalization or emergency department visit for suicide or non-fatal self-harm within 90 days of treatment. For each case, we identified up to fifty controls from the same cohort matched on age, sex, history of self-harm, use of selected psychotropic medications, alcohol abuse and prior admission to a mental health unit. Adjusted odds ratio were used to compare the risk of suicide/self-harm of varenicline to bupropion.

**Results:**

We identified 331 cases and 5,346 matched-controls. Following adjustment for potential confounders, we found that varenicline was not associated with an increased risk of suicide/self-harm relative to bupropion (adjusted odds ratio 1.15; 95% confidence interval 0.71 to 1.87).

**Interpretation:**

Treatment with varenicline does not appear to significantly increase the risk of suicide or self-harm relative to bupropion.

## Introduction

Varenicline is a partial nicotine receptor agonist used for smoking cessation.[1–4] Although it is generally well-tolerated, serious psychiatric adverse events including fatal and non-fatal self-harm have been noted in case-reports and post-marketing reporting.[5, 6] Concerns over these events have prompted the U.S. Food and Drug Administration (FDA) and other regulatory agencies to add warnings about self-harm and suicide to the product labelling and prescribing information of varenicline. However, the association between varenicline use and serious psychiatric adverse events remains unclear.[7] Clinical trials are not powered to detect rare adverse events such as suicide and often exclude patients with psychiatric comorbidies.[1–4, 8] and observational studies have been limited by small numbers of events. Moreover, there have been few studies comparing the risk of suicide between varenicline and bupropion, an antidepressant commonly prescribed for smoking cessation. We sought to examine the association between suicide or non-fatal self-harm and varenicline relative to bupropion using administrative claims databases.

## Methods

We conducted a population-based nested case-control study of Ontario adults (18 years and older) who were dispensed bupropion or varenicline between April 1, 2011 through March 31, 2015. We used Ontario’s administrative databases to ascertain drug exposures and clinical outcomes. Specifically, we ascertained drug exposure using the Ontario Drug Benefit (ODB) database, drug coverage in Ontario is available for all residents with financial needs (due to high drug costs and/or low income) and all residents 65 years of age and older. We identified hospitalizations and emergency department visits using the Canadian Institute for Health Information's Discharge Abstract Database and National Ambulatory Care Reporting System, respectively. These datasets were linked using unique, encoded identifiers, were analyzed at the Institute for Clinical Evaluative Sciences (ICES), and are routinely used to examine drug safety.[9–12]

From within the cohort of patients receiving either varenicline or bupropion, we defined cases as patients with any emergency department (ED) visit or inpatient hospitalization associated with self-harm or suicide (International Classifications of Diseases, 10th edition X60-X84, Y10-Y19, and Y28) who were dispensed one of buproprion or varenicline in the 90 days prior. We restricted our analysis to prescriptions for Zyban® because this is the only formulation of bupropion covered solely as smoking cessation therapy by the Ontario Drug Benefit program. From within the same cohort, we selected up to 50 controls for each case and randomly assigned them an index date over the study period. Controls were eligible if they had no hospitalization or emergency department visits for self-harm/suicide at index, and had been prescribed one (but not both) of the study drugs in the prior 90 days. Controls and cases were matched on age (within 1 year), sex, history of hospitalization or ED visit for self-harm in the previous 5 years, use of any psychotropic medications in the previous 6 months, history of alcohol abuse in the previous 3 years, and admission to a mental health unit in the previous year. The study was approved by the research ethics board of Sunnybrook Health Sciences Centre, Toronto, Ontario, Canada.

We summarized patient characteristics using descriptive statistics and compared cases and controls using standardized differences. We used conditional logistic regression to compare the risk of suicide/self-harm between varenicline and bupropion. We adjusted all models for potential confounders including socioeconomic status, Charlson comorbidity score, health care utilization (number of physicians visits and ED visits in the past year), number of psychiatrist visits (in the past year), past psychotropic use in previous 6 months (antidepressants, antipsychotics, benzodiazepines, mood stabilizers, and stimulants), psychiatric comorbidities, and total number of drugs dispensed in the past 180 days. Analyses were performed using SAS (version 9.2; SAS Institute Inc.).

## Results

We identified 331 cases and 5,346 matched-controls who were treated with varenicline or bupropion 90 days prior to their index date (**Table 1)**. Overall, cases were similar to controls with respect to important demographics and characteristics such as neighbourhood income status, urban status, age, and sex. As expected, cases were more likely to have visited the emergency department, seen a psychiatrist, spent more days in hospital, and had a greater burden of psychiatric morbidity. In our main analysis, we found no increase in risk of suicide/self-harm among varenicline users relative to bupropion users (adjusted odds ratio 1.15; 95% confidence interval 0.71 to 1.87) (**Table 2)**.

**Table 1:** Baseline Characteristics for Cases and Matched Controls.

Characteristics	Cases	Controls	Standardized Difference
	N = 331	N = 5,346	
**Demographic Characteristics:**	** **	** **	** **
Age at index date—Mean (SD)	45.5 (12.1)	45.4 (12.1)	0.01
Male—N (%)	154 (46.5%)	2,541 (46.5%)	0
Income quintile- N (%)			
1	140 (42.3%)	2,274 (44.6%)	0.06
2	84 (25.4%)	1,266 (22.4%)	0.09
3	48 (14.5%)	762 (14.9%)	0.02
4	36 (10.9%)	624 (11.5%)	0.03
5	22 (6.6%)	397 (6.1%)	0.03
Urban residence—N (%)	299 (90.3%)	4,501 (85.7%)	0.19
**Measures of Comorbidity:**	** **	** **	** **
Charlson Comorbidity Score:—N (%)			
No hospitalization	171 (51.7%)	3,865 (64.3%)	0.31
0	91 (27.5%)	705 (18.5%)	0.25
1	37 (11.2%)	381 (10.1%)	0.04
2+	32 (9.7%)	395 (7.0%)	0.11
**Health Care Utilization:**			
Number of visits to ED in the past year—Median (IQR)	2.0 (1.0–6.0)	1.4 (1.0–2.4)	0.44
ED visit or Hospitalization for self-harm in the past 5 years—N (%)	94 (28.4%)	211 (28.4%)	0
Number of visits to a family physician in the past year—Median (IQR)	12.0 (6.0–25.0)	13.1 (9.5–19.5)	0.22
Number of Psychiatrist visits in the past year- N (%)	146 (44.1%)	905 (31.6%)	0.30
Days in hospital in past 1 year *- Mean (SD)	10.0 (16.4)	1.9 (2.8)	0.87
Admission to mental health unit in the past year—N (%)	35 (10.6%)	48 (10.6%)	0
**Psychiatric Disorders (measured in 3 years prior to index date):—**N (%)			
Alcohol Abuse	100 (30.2%)	374 (30.2%)	0
Affective Disorder	113 (34.1%)	629 (23.7%)	0.26
Anxiety or sleep disorder	286 (86.4%)	3,124 (71.2%)	0.50
Psychosis, agitation, and related disorders	98 (29.6%)	496 (19.6%)	0.26
Other mental health disorders	279 (84.3%)	3,353 (74.4%)	0.32
Number of distinct drugs used in past 6 months—Median (IQR)	11.0 (7.0–16.0)	10.2 (7.4–12.3)	0.34
Number of distinct psychotropic medications used in past 6 months—Median (IQR)	3.0 (2.0–5.0)	2.3 (1.9–3.0)	0.50
**Past Medication Use (past 6 months):-** N (%)			
Antidepressants (SSRIs)	257 (77.6%)	3,537 (73.2%)	0.12
Antidepressants (other)	165 (49.8%)	1,256 (36.8%)	0.31
Benzodiazepines	196 (59.2%)	1,906 (42.8%)	0.40
Mood Stabilizers	104 (31.4%)	962 (20.5%)	0.29
Stimulants	21 (6.3%)	160 (5.0%)	0.07

*Note: Days in hospital in past 1 year is calculated only among those had at least 1 day in hospital

**Table 2:** Association of varenicline compared to bupropion and self-harm.

	No. exposed cases (N)	No. Exposed controls(N)	Unadjusted OddsRatio (95% CI)	AdjustedOddsRatio (95% CI)
Varenicline	307	4,764	1.35 (0.86 to 2.11)	1.15 (0.71 to 1.87)
Bupropion (reference)	24	582	1.00	1.00

## Interpretation

In this observational study, we did not find a significantly higher risk of suicide/self-harm with varenicline relative to bupropion use for smoking cessation. To our knowledge, this is the largest study examining this question.

Our findings align with four previously published observational studies and recent FDA reports.[13–16] All observational studies found no increased risk with varenicline use, but confidence in the findings was limited due to smaller sample sizes. Two cohort studies using the UK General Practice Research Data (GPRD) database found similar incidences of fatal and nonfatal self-harm among patients prescribed varenicline or nicotine replacement therapy (0.08% and 0.09%, respectively). A similar incidence was found among bupropion users (0.06%), although a direct comparison with varenicline-treated patients was not made. A 2015 update using the same UK data found similar results with varenicline not being associated with an increased risk of self-harm (adjusted hazard ratio 0.56; (95% confidence interval 0.46 to 0.68).[8] The protective estimate with varenicline may have been due to large differences in baseline characteristics which may have lead to selection bias. A Danish study comparing 17,935 users of varenicline with the same number of bupropion-treated patients found no difference in the incidence of psychiatric adverse events (0.22% vs. 0.26%, respectively), although suicide was not examined as an outcome.[14] Additionally, a meta-analysis of 18 trials submitted to the FDA by the manufacturer of varenicline found no statistically significant excess risk of self-harm compared to placebo. The same meta-analysis found no risk of suicidal ideation, hostility, and psychiatric disorders.[17] Ongoing manufacturer-sponsored clinical trials designed to address this question are scheduled to be published in the upcoming year.[18]

Our study has some limitations that warrant emphasis. First, restricting our analysis to Zyban® may have decreased the number of actual cases associated with off-label use of other bupropion formulations. However, this is unlikely because Zyban® was wholly reimbursed for this indication during our study period. Second, we are unable to identify use of other smoking cessation agents (e.g. nicotine replacement therapy), or the use of non-pharmacological-based smoking cessation programs. Third, we are unable to identify suicides that did not present to the emergency department or hospital and thus may have an underrepresentation of the number of cases in both groups. Lastly, due to the impact of concerns with varenicline and neuropsychiatric side effects, there may be increased aversion to prescribing varenicline to those at higher risk of self-harm. However, we believe that this aversion can be extended to both medications and thus would not impact our findings.

In conclusion, varenicline was not associated with a higher risk of suicide/self-harm relative to bupropion. Although our findings are not meant to endorse preferential use of varenicline, our study reinforces the prevailing view that varenicline does not impart an excess risk of suicide relative to other smoking cessation therapies. Future clinical trials are important to clarify the comparative safety of varenicline but we urge the importance of observational work such as our study to give a balanced view of real-world safety.
